# MRI-based Brain Healthcare Quotients: A bridge between neural and behavioral analyses for keeping the brain healthy

**DOI:** 10.1371/journal.pone.0187137

**Published:** 2017-10-27

**Authors:** Kiyotaka Nemoto, Hiroki Oka, Hiroki Fukuda, Yoshinori Yamakawa

**Affiliations:** 1 Department of Neuropsychiatry, Division of Clinical Medicine, Faculty of Medicine, University of Tsukuba, Tsukuba, Ibaraki, Japan; 2 ImPACT Program of Council for Science, Technology and Innovation (Cabinet Office, Government of Japan), Chiyoda, Tokyo, Japan; Nathan S Kline Institute, UNITED STATES

## Abstract

Neurological and psychiatric disorders are a burden on social and economic resources. Therefore, maintaining brain health and preventing these disorders are important. While the physiological functions of the brain are well studied, few studies have focused on keeping the brain healthy from a neuroscientific viewpoint. We propose a magnetic resonance imaging (MRI)-based quotient for monitoring brain health, the Brain Healthcare Quotient (BHQ), which is based on the volume of gray matter (GM) and the fractional anisotropy (FA) of white matter (WM). We recruited 144 healthy adults to acquire structural neuroimaging data, including T1-weighted images and diffusion tensor images, and data associated with both physical (BMI, blood pressure, and daily time use) and social (subjective socioeconomic status, subjective well-being, post-materialism and Epicureanism) factors. We confirmed that the BHQ was sensitive to an age-related decline in GM volume and WM integrity. Further analysis revealed that the BHQ was critically affected by both physical and social factors. We believe that our BHQ is a simple yet highly sensitive, valid measure for brain health research that will bridge the needs of the scientific community and society and help us lead better lives in which we stay healthy, active, and sharp.

## Introduction

Various mental illnesses currently affect millions of people, and enormous social and economic resources are spent to treat them [1]. Huge neuroscientific projects are therefore being conducted in the U.S. [2], Europe [3], and other countries, where structural and functional neuroimaging techniques are widely used to reveal physiological functions of the central nervous system. The brain-behavior relationship has thus been gradually delineated for a wide range of mental illnesses, including neurological disorders (e.g., Alzheimer's disease [4] and Parkinson's disease [5]), psychiatric disorders (e.g., depressive disorder [6] and schizophrenia [7]) and developmental disorders (e.g., autism spectrum disorder [8]). Most of these studies have compared the brain structures or functions of patients with specific diagnoses with “healthy” participants.

Here, “healthy” simply refers to the state of participants who do not have such mental illnesses. However, as described in the constitution of the WHO [9], “health is a state of complete physical, mental and social well-being and not merely the absence of disease or infirmity.” Maintaining a healthy state is preferable and important, in terms of both improvement of the quality of life (QOL) and reduction of medical expenses for individuals and society. Despite these substantial implications, few neuroscientific studies have focused on how we can measure and maintain brain health. As a first step for this line of research, we advocate that establishing a quotient for brain health is a necessary foundation for future human neuroscience studies. Making an index for brain healthcare and clarifying the factors associated with it would help bridge the needs of both the scientific community and society.

A good state of brain health is undoubtedly determined by the cellular structure of gray (GM) and white (WM) matter. In GM, an appropriate amount of expanse of dendrites and a reasonable increase in synapses of the neural cells are thought to be signs of a good state of health [10]. This good health induces high plasticity in synapses and can be interpreted as indicating flexibility of learning in the future [11]. This brain state is reflected in the GM volume [12]. In addition, WM plasticity is influenced by various factors such as fiber organization, myelin formation, myelin remodeling, changes in oligodendrocyte or astrocyte, and angiogenesis [13]. The transmission efficiency of the network between brain regions is thus supported by WM integrity, which is reflected in the fractional anisotropy (FA) of axons [14], as measured by diffusion tensor imaging (DTI).

Considering these observations, we propose an index for brain healthcare, the Brain Healthcare Quotient (BHQ), which includes two subordinate indices: the GM-BHQ based on the volume of GM, as assessed by voxel-based morphometry (VBM), and the FA-BHQ (WM-BHQ) based on the FA value of WM, as assessed by DTI. Here we provide evidence that the BHQs are sensitive to age-related decline, specifically, to decreases in GM volume and WM integrity. We also demonstrate that physical and social factors, which may affect brain health, significantly impact the BHQs.

## Materials and methods

### Subjects

One hundred and forty-four healthy participants (64 females and 80 males), aged 25–69 (mean (M) ± standard deviation (SD): 48.4 ± 8.1 years old), were recruited in local cities in Hyogo, Kyoto, and Tokyo, Japan. This study was approved by the ethics committees of RIKEN (approval number KOBE-IRB-15-13), Kyoto University (approval number 27-P-13), and the University of Tokyo (approval number 402) and was performed in accordance with the guidelines and regulations of these research institutions. All participants gave written informed consent prior to participation, and participant anonymity has been preserved. While recruiting participants, those who had medical histories of neurological, psychiatric or medical conditions that could potentially affect the central nervous system were excluded.

### MRI data acquisition

All magnetic resonance imaging (MRI) data were collected using a 3-T Siemens scanner (Verio, Siemens Medical Solutions, Erlangen, Germany or MAGNETOM Prisma, Siemens, Munich, Germany) with a 32-channel head array coil at RIKEN, Kyoto University, and the University of Tokyo.

A high-resolution structural image was acquired using a three-dimensional (3D) T1-weighted magnetization-prepared rapid-acquisition gradient echo (MP-RAGE) pulse sequence. The parameters were as follows: repetition time (TR), 1900 ms; echo time (TE), 2.52 ms; inversion time (TI), 900 ms; flip angle, 9°; matrix size, 256 × 256; field of view (FOV), 256 mm; slice thickness, 1 mm.

DTI data were collected with spin-echo echo-planar imaging (SE-EPI) with GRAPPA (generalized autocalibrating partially parallel acquisitions). The image slices were parallel to the orbitomeatal (OM) line. The parameters were as follows: TR, 14100 ms; TE, 81 ms, flip angle, 90°; matrix size, 114 x 114; FOV, 224 mm; slice thickness, 2 mm. A baseline image (b = 0 s/mm^2^) and 30 different diffusion orientations were acquired with a b value of 1000 s/mm^2^.

### MRI data analysis

T1-weighted images were preprocessed and analyzed using Statistical Parametric Mapping 12 (SPM12; Wellcome Trust Centre for Neuroimaging, London, UK) running on MATLAB R2015b (Mathworks Inc., Sherborn, MA, USA), where the preprocessing steps of segmentation, bias correction, and spatial normalization are incorporated into a single generative model. Each MPRAGE image was segmented into GM, WM, and cerebrospinal fluid (CSF) images using SPM12 prior probability templates. The intensity non-uniformity bias correction was applied to aid segmentation by correcting for scanner-induced smooth intensity differences that varied in space. Subsequently, the segmented GM images were spatially normalized using the diffeomorphic anatomical registration through exponentiated lie algebra (DARTEL) algorithm [15]. A modulation step was also incorporated into the preprocessing model to reflect regional volume and preserve the total GM volume from before the warp. As a final preprocessing step, all normalized, segmented, modulated images were smoothed with an 8-mm full width at half-maximum (FWHM) Gaussian kernel.

Additionally, the global volumes of GM, WM, and CSF for each scan were calculated. The volume of each tissue class was estimated as the total number of voxels multiplied by the voxel size. Intracranial volume (ICV) was calculated by summing the GM, WM, and CSF images for each subject. Proportional GM images were generated by dividing smoothed GM images by ICV to control for differences in whole-brain volume across participants. Using these proportional GM images, mean and standard deviation (SD) images were generated from all participants. Next, we calculated the GM brain healthcare quotient (BHQ), which is similar to the intelligence quotient (IQ). The mean value was defined as BHQ 100 and SD was defined as 15 BHQ points. By this definition, approximately 68% of the population is between BHQ 85 and BHQ 115, and 95% of the population is between BHQ 70 and BHQ 130. Individual GM quotient images were calculated using the following formula: 100 + 15 × (individual proportional GM—mean) / SD. Regional GM quotients were then extracted using an automated anatomical labeling (AAL) atlas [16] and averaged across regions to produce participant-specific GM-BHQs.

DTI data were preprocessed using FMRIB Software Library (FSL) 5.0.9 [17]. First, all diffusion images were aligned with the initial b0 image, and motion correction and eddy current distortion correction was performed using eddy_correct. Following these corrections, FA images were calculated using dtifit. FA images were then spatially normalized into the standard Montreal Neurological Institute (MNI) space using FLIRT and FNIRT. FLIRT, a linear registration tool, was used to roughly align a set of brains to MNI space. Then FNIRT, a non-linear registration tool, was used to achieve better registration. After spatial normalization we smoothed the data with an 8-mm FWHM. Mean and SD images were generated from all the FA images, and both individual FA quotient images and GM-BHQ images were calculated. Individual FA quotient images were calculated using the following formula: 100 + 15 × (individual FA–mean) / SD. Regional FA quotients were extracted using Johns Hopkins University (JHU) DTI-based white-matter atlases [18] and averaged across regions to produce participant-specific FA-BHQs.

### Physical factors

#### Body mass index (BMI)

Participants were classified into 3 groups: obesity (BMI≥25, n = 24, 16.7%), emaciation (BMI<18.5, n = 15, 10.4%), and normal weight (18.5≤BMI<25, n = 105, 72.9%) using the criteria of the Japan Society for the Study of Obesity [19].

#### Blood pressure

Participants were classified into 3 groups: hypertension (n = 32, 22.2%), hypotension (n = 14, 9.7%), and normal blood pressure (n = 98, 68.1%). To define hypertension, we followed the WHO criteria (above 140/90 millimeters). To define hypotension, we followed the practical criteria in Japan (systolic blood pressure less than 100 millimeters) because the WHO does not provide medical or official criteria for hypotension.

#### Pulse

We also measured the pulse of all participants (M = 78.8, SD = 10.83).

#### Daily time use

Participants answered questions about how much time they spent doing various activities on typical weekdays and holidays. From their responses, we selected variables for which the mean values were above 0.5 hr. For weekday activities, sleep, work, housework, meals, personal business, attending work or school, rest and relaxation, and TV/radio/newspaper/magazine were selected. For holiday activities, traveling time other than commuting to work or school, shopping, sports, hobbies, and companionship were selected.

### Social factors

#### Subjective socioeconomic status

We measured two aspects of subjective socioeconomic status: stratum identification and financial worries. For stratum identification, participants were asked “How do you rate your standard of living compared to the general public?” Responses were given on a 5-point scale: “highest” = 5, “upper-middle” = 4, “middle” = 3, “lower-middle” = 2, and “lowest” = 1 (M = 3.1, SD = 0.80, n = 141). For financial worries, participants were asked “Do you have worries and anxiety about your present or future income and assets?” Responses were “yes (n = 49, 34.0%)” or “no (n = 95, 66.0%)”.

#### Subjective well-being

We measured two aspects of subjective well-being: life satisfaction and life improvement. For life satisfaction, participants were asked “To what extent are you satisfied with your current life?” Responses were given on a 4-point scale from “satisfied” = 4 to “not satisfied” = 1 (M = 2.8, SD = 0.70, n = 136). For life improvement, participants were asked two questions. The first question was “How is your life now compared to this time last year?” Responses were given on a 3-point scale: “got better” = 3, “the same” = 2, and “got worse” = 1). The second question was “How do you think your life will be in the future?” Responses were given on a 3-point scale: “get better” = 3, “the same” = 2, and “get worse” = 1). The answer for these two questions were added, and the correlation coefficients were r = 0.469, with p < 0.001 (M = 4.03, SD = 0.84, n = 132).

#### Post-materialism

Participants were asked, “Which do you think should be prioritized from now on, richness of the mind and heart or material and economic richness?” Responses were given on a 3-point scale: “priority to richness of the mind and heart” = 3, “don’t know” = 2, and “priority to material and economic richness” = 1 (M = 2.32, SD = 0.80, n = 142).

#### Epicureanism and asceticism

Participants were asked, “Which do you think should be prioritized from now on, enjoying your present life or preparing for the future?” Responses were chosen from among “priority to enjoying present life,” “don’t know,” or “priority to preparing for the future.” Because this variable had a non-linear association with the GM-BHQ and FA-BHQ, we used it as a categorical variable and made a dummy variable (n = 140). When the answer was “priority to enjoying present life” (n = 39, 27.9%), the dummy variable was “Epicureanism = 1” and other responses were “Epicureanism = 0,” and when the response was “priority to preparing for the future” (n = 58, 41.4%), the dummy variable was “asceticism = 1” and other responses were “asceticism = 0.” The reference group was “don’t know” (n = 43, 30.7%).

### Statistical analysis

First, the correlation coefficient between BHQ (GM-BHQ and FA-BHQ) and age were examined. Then, in order to investigate the relationships of physical factors on BHQ, general linear regression analyses were used. We employed three models for analysis: Model 1.1 assessed the relationship of BMI with BHQ, adjusting for age and sex; Model 1.2 then introduced blood pressure and pulse as an additional independent variable to Model 1.1. Model 1.3 introduced daily time use as an additional independent variable to Model 1.2. In model 1.3, independent variables were selected by the stepwise method because there were many variables of daily time use. We added these respective variables to the models based on the hypotheses that blood pressure is more closely related to BHQ than is BMI, and that daily time use is more closely related to BHQ than are the previous two variables. We also investigated the relationship between BHQ and social factors in a similar way using general linear regression analyses. For these analyses, we employed another set of models: Model 2.1 assessed the relationship of socioeconomic status with BHQ after adjusting for age and sex; Model 2.2 then introduced subjective well-being as an additional independent variable to Model 2.1; Model 2.3 introduced attitude (post-materialism and Epicureanism) as an additional independent variable to Model 2.2. Similarly to the physical factors of the first model series, we added these variables based on the hypotheses that subjective well-being is more closely related to BHQ than is socioeconomic status, and that attitude is more closely related to BHQ than are the previous two variables. The significance level was determined at p < .05. All statistical analyses were conducted using IBM SPSS Statistics Version 20 (IBM Corp., Armonk, NY, USA).

## Results

### BHQ and age

**Fig 1:**
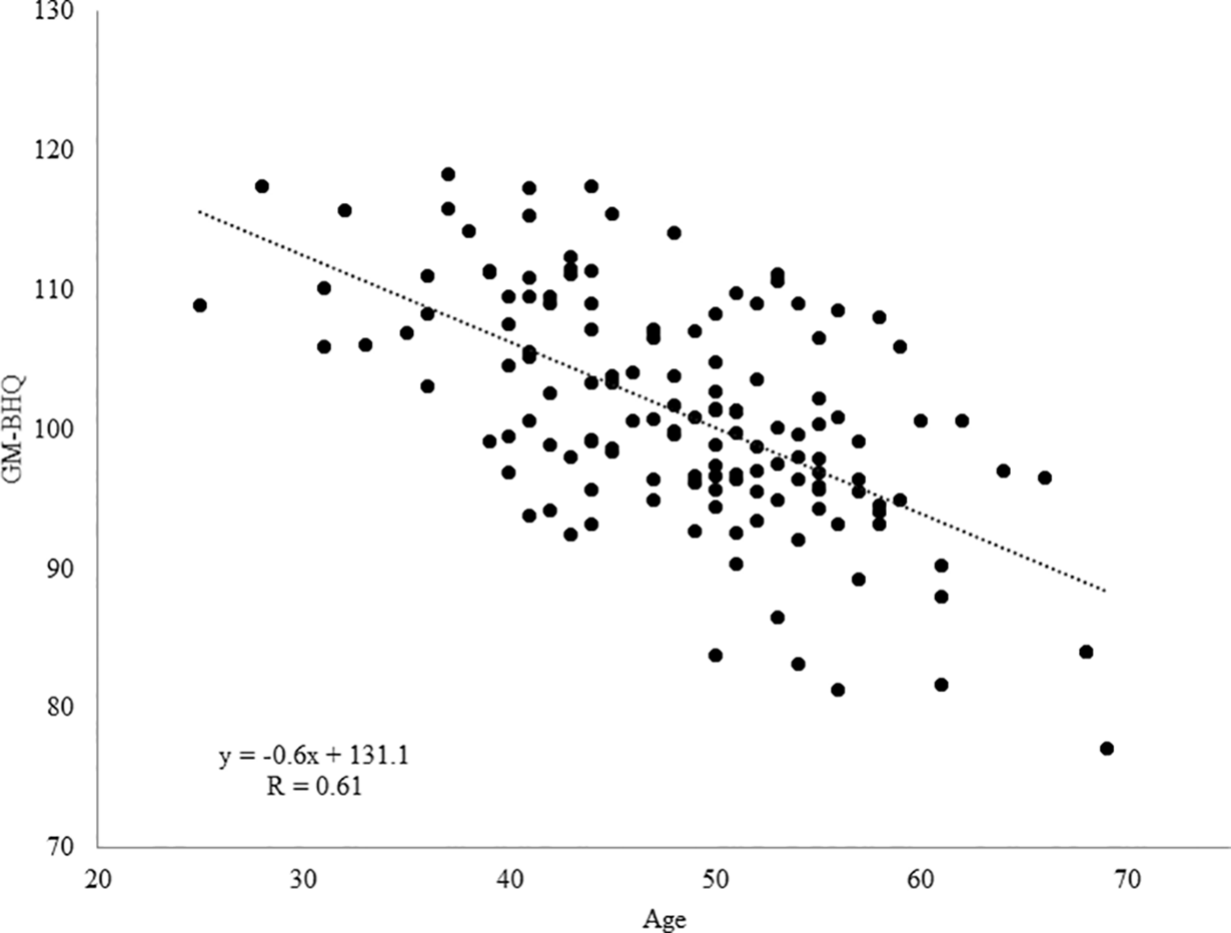
Scatter plot and regression line of age on GM-BHQ. We found a negative correlation between GM-BHQ and age (n = 144, R = 0.610, b = -0.618, p < 0.001).

**Fig 2:**
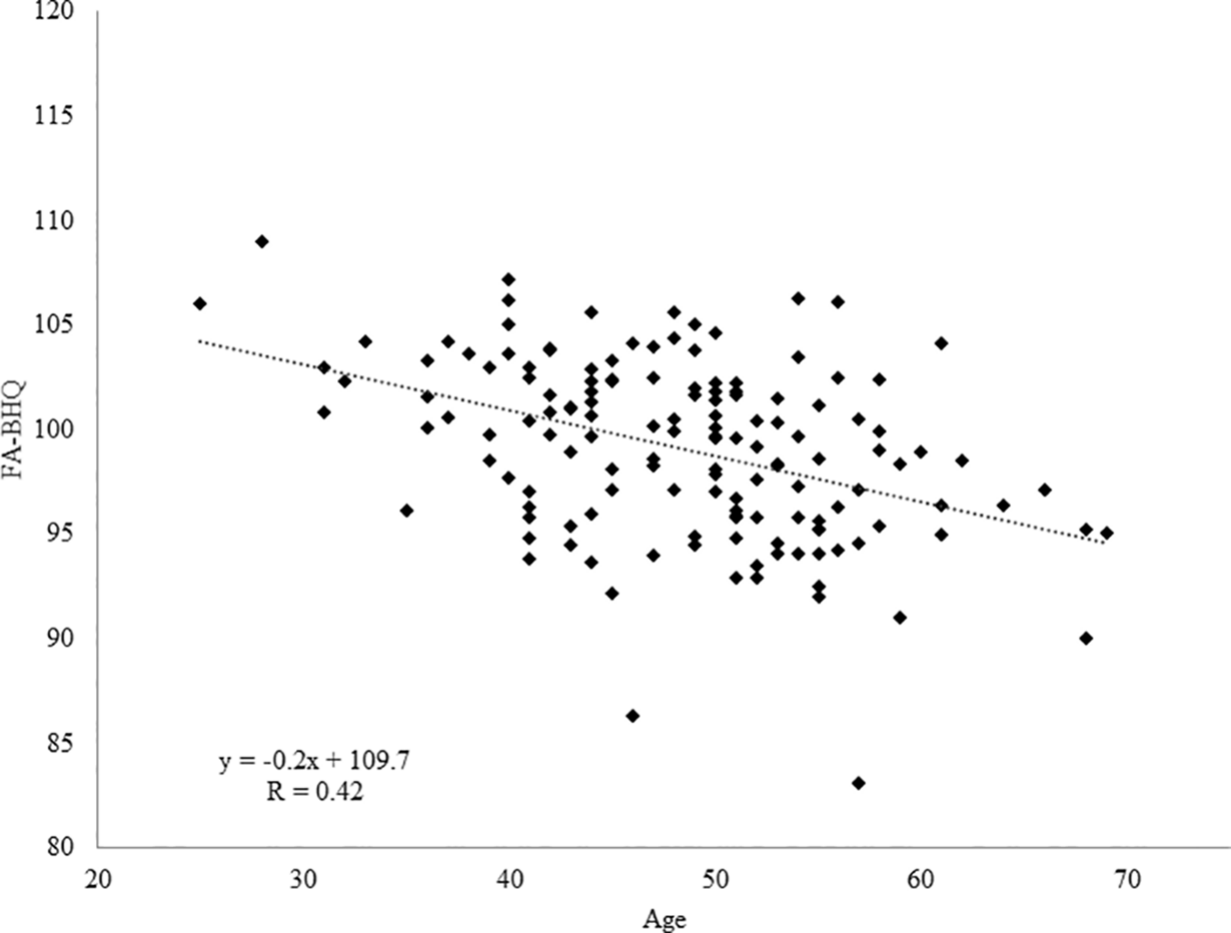
Scatter plot and regression line of age on FA-BHQ. We found a negative correlation between FA-BHQ and age (n = 144, R = 0.417, b = -0.219, p < 0.01).

To confirm whether the GM-BHQ and FA-BHQ reflect age-related declines, we examined the association between the BHQs and age. The results are illustrated in Figs 1 and 2. We found a negative correlation between GM-BHQ and age (n = 144, R = 0.610, b = -0.618, p < 0.001). Additionally, we found a negative correlation between FA-BHQ and age (n = 144, R = 0.417, b = -0.219, p < 0.001). Note that previous studies have shown that neuroimaging results are not confounded by scanner differences in a multi-site study, thus allowing the pooling of data obtained from different scanners [20–24]. In fact, the present results remained virtually unchanged even if we included the factor of scanner differences as a nuisance variable (GM-BHQ: R = 0.683, b = -0.544, p < 0.001; FA-BHQ: R = 0.618, b = -0.158, p < 0.001).

Then, to determine the most appropriate model to estimate age, we conducted hierarchical multiple regression analysis with the outcome of age and compared the coefficients of determination (R^2^) of each model: GM-BHQ, FA-BHQ, or both GM-BHQ and FA-BHQ. As a result, we found coefficients of determination (R^2^) were significantly increased when both GM-BHQ and FA-BHQ were used as independent variables compared with when GM-BHQ and FA-BHQ were used separately (GM-BHQ: R^2^ = 0.372, FA-BHQ: R^2^ = 0.174, both GM-BHQ and FA-BHQ: R^2^ = 0.443; ΔR^2^ = 0.071 and 0.269, respectively, both p < 0.001). Thus, 44.3% of the variance of the dependent variable (age) is explained when both GM-BHQ and FA-BHQ are used as independent variables. These effects did not vary by the respondent’s sex (male: n = 80, R = 0.681, GM-BHQ b = -0.654, p < 0.001, FA-BHQ b = -0.481, p = 0.004; female: n = 64, R = 0.667, GM-BHQ b = -0.595, p < 0.001, FA-BHQ b = -0.615, p = 0.002).

### BHQ and physical factors

To investigate which physical factors contribute to keeping the brain healthier, we examined the association between the BHQs and the following physical factors: BMI, blood pressure, and daily time use. Since FA is prone to be non-normally distributed, we first verified that the residual of each model regarding FA-BHQ was normally distributed. Shapiro-Wilk coefficients for each model were 0.982 (p = 0.058) for Model 1.1, 0.985 (p = 0.123) for Model 1.2, and 0.987 (p = 0.220) for Model 1.3, which confirmed the normal distribution of residuals. Table 1 shows the results of multiple regression analysis with the outcome of GM-BHQ and FA-BHQ according to these factors, adjusting for age and sex. We found that obesity was significantly associated with a lower GM-BHQ (R = 0.746, b = -2.761, p = 0.030) and that hypotension was significantly associated with a lower FA-BHQ (R = 0.505, b = -2.830, p = 0.015). With regard to daily time use on weekdays, long rest and relaxation times were associated with greater GM-BHQ (R = 0.799, b = 0.990, p = 0.006). Long mealtimes was associated with greater FA-BHQ (R = 0.543, b = 1.164, p = 0.013). On holidays, a long time spent on personal business (R = 0.799, b = 1.207, p = 0.002), short mealtimes (R = 0.799, b = -1.272, p = 0.006), and short rest and relaxation times (R = 0.799, b = -0.454, p = 0.030) were associated with greater GM-BHQ. Long traveling time was associated with greater FA-BHQ (R = 0.543, b = 0.701, p = 0.035). Data used for the analysis is provided in S1 Data.

**Table 1:** Multiple regression analysis of physical factors on BHQ.

	Model 1.1				Model 1.2				Model 1.3^a^			
	GM-BHQ		FA-BHQ		GM-BHQ		FA-BHQ		GM-BHQ		FA-BHQ	
	b^b^	p-value	b	p-value	b	p-value	b	p-value	b	p-value	b	p-value
Age	-0.531	< 0.001***	-0.210	< 0.001***	-0.534	< 0.001***	-0.229	< 0.001***	-0.542	< 0.001***	-0.231	0.001***
Sex (male = 1, female = 2)	6.539	< 0.001***	1.129	0.101	6.320	< 0.001***	1.371	0.044*	5.074	< 0.001***	1.126	0.077
BMI												
obesity (BMI ≥ 25.0)	-2.761	0.03*	0.598	0.496	-2.306	0.085	0.814	0.371	-2.097	0.071	-	
emaciation (BMI < 18.5)	0.451	0.777	-1.695	0.127	0.322	0.844	-1.270	0.256	-		-	
Blood pressure												
hypertension	-		-		-1.979	0.103	-0.534	0.517	-		-	
hypotension	-		-		-0.114	0.946	-2.830	0.0150*	-		-2.822	0.01*
Pulse	-		-		0.059	0.176	-0.048	0.108	-		-	
Daily time use												
weekday: rest	-		-		-		-		0.990	0.006**	-	
weekday: housework	-		-		-		-		0.418	0.063	-	
weekday: meal	-		-		-		-		-		1.164	0.013*
holiday: personal business	-		-		-		-		1.207	0.002**	-	
holiday: meal	-		-		-		-		-1.272	0.006**	-	
holiday: rest	-		-		-		-		-0.454	0.03*	0.259	0.072
holiday: travel	-		-		-		-		-		0.701	0.035*
R	0.746	< 0.001***	0.448	< 0.001***	0.754	< 0.001***	0.505	< 0.001***	0.799	< 0.001***	0.543	< 0.001***
R^2^	0.556		0.200		0.569		0.255		0.639		0.295	

n = 144

*p < 0.05

**p < 0.01

***p < 0.001

a In Model 3, independent variables were selected by the stepwise method.

b Regression coefficient

### BHQ and social factors

To investigate which social factors are associated with brain health, we conducted multiple regression analysis between the BHQs and the following social factors after adjusting for age and sex: 1) subjective socioeconomic status, including stratum identification and financial worries; 2) subjective well-being, including life satisfaction and sense of life improvement; 3) post-materialism [25], in which priority is given to richness of mind and heart rather than material and economic richness; and 4) Epicureanism, in which priority is given to living for the moment rather than preparing for the future. As we did for FA-BHQ and the physical factors, here we also verified that the residual of each model regarding FA-BHQ was normally distributed. Shapiro-Wilk coefficients for each model were 0.990 (p = 0.474) for Model 2.1, 0.990 (p = 0.528) for Model 2.2, and 0.993 (p = 0.802) for Model 2.3, which confirmed the normal distribution of residuals. The results are summarized in Table 2. We found that high stratum identification was significantly associated with greater GM-BHQ (R = 0.748, β = 0.162, p = 0.015), and a sense of life improvement was significantly associated with greater FA-BHQ (R = 0.476, β = 0.221, p = 0.020). Post-materialism was significantly associated with a greater FA-BHQ (R = 0.507, β = 0.184, p = 0.044). Data used for the analysis is also provided in S1 Data.

**Table 2:** Multiple regression analysis of social factors on BHQ.

	Model 2.1				Model 2.2				Model 2.3			
	GM-BHQ		FA-BHQ		GM-BHQ		FA-BHQ		GM-BHQ		FA-BHQ	
	β^a^	p-value	β	p-value	β	p-value	β	p-value	β	p-value	β	p-value
Age	-0.546	< 0.001***	-0.420	< 0.001***	-0.576	< 0.001***	-0.375	< 0.001***	-0.567	< 0.001***	-0.382	< 0.001***
Sex (male = 1, female = 2)	0.435	< 0.001***	0.044	0.606	0.436	< 0.001***	0.062	0.465	0.454	< 0.001***	0.081	0.343
Subjective socioeconomic status												
stratum identification	0.162	0.015*	0.084	0.348	0.208	0.003**	0.026	0.781	0.188	0.008**	-0.014	0.881
financial worries^b^	0.041	0.536	0.031	0.727	0.019	0.772	0.034	0.709	0.033	0.634	0.084	0.367
Subjective well-being												
life satisfaction					-0.081	0.243	-0.031	0.740	-0.080	0.252	-0.051	0.591
life improvement					-0.095	0.176	0.221	0.020*	-0.070	0.321	0.249	0.010*
Post-materialism									0.077	0.249	0.184	0.044*
Epicureanism^c^									-0.126	0.093	-0.044	0.661
Asceticism									-0.050	0.510	-0.049	0.633
R	0.748	< 0.001***	0.434	< 0.001***	0.759	< 0.001***	0.476	< 0.001***	0.769	< 0.001**	0.507	< 0.001***
R^2^	0.559		0.189		0.576		0.227		0.592		0.257	

n = 123

*p < 0.05

**p < 0.01

***p < 0.001

a Standardized regression coefficient

b Having worries and anxiety about present or future income and assets = 1, everything else = 0.

c Because a non-linear association with BHQ was shown, we used this variable as a categorical variable (Epicureanism/asceticism/don’t know).

The reference group was “don’t know.”

## Discussion

In the present study, we proposed an MRI-based quotient, the BHQ, for monitoring brain health based on the volume of GM and the FA of WM. The results showed that the BHQ is sensitive to age-related decline in GM volume and WM integrity. Further analysis revealed that the BHQ is affected by both physical and social factors, indicating the validity of the BHQ as a potential measure for brain health. We believe that our BHQ is a simple yet highly sensitive tool for brain-health research that will bridge the needs of the scientific community and society.

Since BHQ is a single score derived from GM volume or FA values, it does not provide any information about the connectivity of local brain regions. Nevertheless, BHQ could provide a simple index that may help laypeople grasp the state of their brains in terms of grey matter volume or integration of white matter, and motivate them toward healthier lifestyles. However, for the practical implementation of this method, it is necessary to establish a sufficiently large database because we must determine whether BHQ is related to various factors such as neurological/psychiatric disorders or neurocognitive functions. With the availability of such information, it would be easy for anyone to understand the health status of their own brain.

We found that the GM-BHQ and FA-BHQ were negatively correlated with age, confirming that both quotients are highly sensitive to age-related declines in the brain. The GM-BHQ and FA-BHQ were significant predictors of age, even when the separate analyses were conducted for men and women. Critically, the combination of GM-BHQ and FA-BHQ accounted for more than 40% of the variance in age. Our results are in accordance with a previous study that revealed that aging affects brain structure and its functions [26]. In this study, we investigated the relationship between BHQ and age to show the validity of the BHQ, and previous studies have reported a relationship between brain structure and general intelligence [27,28]. However, further study is needed to reveal whether BHQ is related to general intelligence.

The results demonstrated that the BHQs were affected by both physical and social factors that cannot be explained by age, indicating that the BHQs are potentially valid measures for brain health. Among physical factors, we found that obesity and hypotension were associated with lower GM-BHQ and FA-BHQ, respectively. These results suggest that ameliorating metabolic syndrome and preventing hypotension are important at both a physical and a neural level. These results are consistent with previous studies revealing the association between brain structure and obesity [29–31], metabolic syndrome [32], and blood pressure [33]. Similar to several physical diseases, such as diabetes mellitus and heart disease [34,35], obesity and abnormal blood pressure increase the risk of harming brain health.

In addition to these physical factors, we also found that lifestyle is a critical factor affecting the BHQs, raising the possibility that moderately controlling lifestyle is important for maintaining brain health. Specifically, long rest times and long mealtimes on weekdays were positively associated with the GM-BHQ and the FA-BHQ, respectively, indicating that taking time to relax and have a long meal on weekdays has positive effects on GM volume and WM integrity. By contrast, long rest times and long mealtimes on holidays were negatively associated with the GM-BHQ. Although we do not know the precise reason for these seemingly contradictory results, one possible interpretation is that spending too much time relaxing on holidays might have negative effects on GM volume. Instead of resting for too long on holidays, doing various activities such as conducting personal business, doing housework, or travelling might be better for brain health. Consistent with the present results, the association between brain health and lifestyle has been suggested by the literature [36].

Consistent with the previous studies that have identified associations between brain structure and social factors (socioeconomic status [37], psychological traits [38], and attitudes [39,40]), we found that the BHQs were associated with stratum identification, sense of life improvement, post-materialism and Epicureanism. Consistent with epidemiological studies that revealed positive correlations between high subjective socioeconomic status and good physical health regardless of objective income and education [41,42], this study revealed that the self-perception of having a high social status had positive effects on GM volume regardless of whether the participant had financial worries. Similarly, in addition to psychological studies showing associations between subjective well-being and various outcomes such as physical health, future success and longevity [43,44], this study showed that a sense of life improvement (a sense of the participant’s life getting better) had positive effects on WM integrity. Furthermore, post-materialism (priority given to richness of mind and heart rather than material and economic richness) was positively correlated with the FA-BHQ. These results suggest that mature values and a careful way of thinking about the future might have positive effects on brain health. To make it clear how BHQ is related to social factors, questionnaires such as Socio Economic Status Scale [45], Social Adaptation Self-evaluation Scale (SASS) [46], or the Sheehan Disability Scale (SDISS or SDS) [47] might be useful. These results also suggest that the BHQs are sensitive not only to physical health but also to social and psychological health, which is consistent with many previous neuroscientific studies that have shown an overlap between the brain mechanisms for processing the physical environment and the social environment [48,49].

The GM-BHQ and FA-BHQ yielded somewhat different patterns of results. The GM-BHQ can be interpreted as being associated with relatively stable and holistic factors: socioeconomic status, activities such as personal business, and a balanced lifestyle (i.e., neither too busy nor too idle). In contrast, the FA-BHQ can be interpreted as being associated with relatively short-term and inconstant psychological factors such as a sense of life improvement and priority to richness of mind, and with a series of behaviors that seem to reduce stress: taking leisurely meals on weekdays and going out on holidays. We speculate that the GM-BHQ and FA-BHQ have differential influences on the physical and social factors, although further studies are needed to determine whether some or all of the present results can be replicated.

Two further limitations of the present study warrant attention. First, a quotient based on GM volume and FA value might not be sufficient to capture the complexity of the brain and the variability that exists across individuals. While we focused on brain structure in this study, recent studies have shown that signals obtained using functional MRI (fMRI), including resting-state fMRI, could be a promising index to evaluate how well the brain network is functioning [50]. By integrating resting-state fMRI into the BHQ, we might be able to monitor shorter-term changes in the brain, such as changes caused by momentary worries or satisfaction. Second, we have not compared the BHQs of healthy participants with those of patients with neurological or psychiatric disorders. We need to test whether the BHQs satisfactorily reflect the status of brain health using an independent dataset, especially the data obtained from individuals with mental illnesses. Despite these limitations, we believe that the BHQs represent an important step toward promoting brain health research. Future studies exploring the relationship between BHQ and various factors, such as vital data, lifestyle, psychological state, or social cognition, might help us live better lives during which we stay healthy, active, and sharp.

## Supporting information

S1 Data Data used for regression analyses of physical and social factors. (XLSX)
